# Mutation spectrum of *CYP1B1* in North Indian congenital glaucoma patients

**Published:** 2009-06-13

**Authors:** Mukesh Tanwar, Tanuj Dada, Ramanjit Sihota, Taposh K. Das, Usha Yadav, Rima Dada

**Affiliations:** 1 Laboratory for Molecular Reproduction and Genetics, Department of Anatomy, AIIMS, New Delhi, India; 2 Dr. R.P. Centre for Ophthalmic Sciences, AIIMS, New Delhi, India; 3 Electron Microscope Facility, AIIMS, New Delhi, India; 4 Guru Nanak Eye Centre and Maulana Azad Medical College, Bahadur Shah Zafar Marg, New Delhi, India

## Abstract

**Purpose:**

Mutations in Cytochrome P450 (CYP1B1) are a predominant cause of congenital glaucoma. This study was planned with the aim to identify the mutation profile of *CYP1B1* in North Indian primary congenital glaucoma (PCG) patients.

**Methods:**

After ethical clearance, 50 congenital glaucoma patients and 50 ethnically matched controls were recruited in this study. Genomic DNA was isolated from the blood and trabecular meshwork, and *CYP1B1* was screened for the six most prevalent mutations (termination at 223 [Ter@223], Gly61Glu, Pro193Leu, Glu229Lys, Arg368His, and Arg390Cys) by polymerase chain reaction-restriction fragment length polymorphism (PCR-RFLP). DNA sequencing was done to identify other mutations and for confirmation of RFLP positive samples.

**Results:**

On PCR-RFLP, 21/50 cases (42%) were found positive for one or more of these mutations. However, on sequencing, we found that 23/50 (46%) harbored the *CYPIB1* mutations. Ter@223 was found in 18%, p.R390H in 16%, and p.R368H in 8% of cases. Three novel mutations, p.L24R, p.F190L, and p.G329D, were identified by DNA sequencing. Leucine, phenylalanine, and glycine are conserved at the 24th, 190th, and 329th position in the CYP1B1 protein in different species, suggestive of important functions at these loci. Ter@223 was found to be the most prevalent mutation in our patients while p.R368H was most prevalent in southern India. The difference in frequency and mutation profile may be due to the heterogeneous Indian population. Pathogenic *CYP1B1* mutations impair anterior chamber development and differentiation by blocking the aqueous outflow and raising intraocular pressure (IOP).

**Conclusions:**

Three novel mutations were identified in this study. Studies of pathogenic sequence variants in *CYP1B1* in different populations may contribute to a better understanding of the disease pathogenesis. This may lead to the development of novel therapeutic approaches in the near future.

## Introduction

Primary congenital glaucoma (PCG; OMIM 231300; provided in the public domain by the National Center for Biotechnology Information, Bethesda, MD) is an inherited ocular disorder of the trabecular meshwork and anterior chamber angle. This leads to the impairment of aqueous outflow, increased intraocular pressure (IOP), and optic nerve damage that can result in permanent vision loss. The term PCG is reserved for those cases in which the only anatomic defect observed is isolated trabeculodysgenesis. It is the second largest cause of blindness, accounting for 15% of cases of blindness. The disease manifests in the neonatal or early infantile period with symptoms of enlargement of the globe, opacification of the cornea, and breaks in Descemet’s membrane [1]. It is characterized by the loss of retinal ganglion cells which leads to irreversible blindness. It is bilateral in 80% of cases. More than 80% of cases present within first year of life out of which the disease is diagnosed in the neonatal period in 25% of the cases and within the first six months of life in 60% of cases. PCG accounts for 55% of primary pediatric glaucoma and is the most common type. Its expression and penetrance varies from 40% to 100%. The prevalence of PCG varies across ethnic communities ranging from 1 in 10,000–20,000 in the western populations [2] to 1 in 2,500 and 1 in 1,250 in the Saudi Arabian population [3] and Gypsy population of Slovakia [2], respectively. In the Indian state of Andhra Pradesh, prevalence is 1 in 3,300 [4]. The high incidence in the eastern countries is thought to be due to consanguineous marriages. Early and reliable diagnosis of the disease is vital, so that appropriate and prompt medical and surgical interventions can be initiated. This could in turn prevent visual loss and save vision of the child.

Although there has been much progress in finding new genes and detecting disease-related mutations, little is known about the function of the mutated gene products and the underlying pathogenic mechanisms. Further, it is estimated that all the known loci/genes of glaucoma account for the minority of total cases of glaucoma, and hence, many other genes remain to be identified. Even though three different loci have been mapped for PCG [5,6], mutations in Cytochrome P450 (*CYP1B1*; GLC3A) are the most predominant cause of disease [7] and are reported in various ethnic backgrounds [7-10]. An additional PCG locus, GLC3B [6], has been mapped to the short arm of chromosome 1 and a third locus, GLC3C, to 14q24.3, but these genes have not been identified. Recently, studies showed the association of *CYP1B1* with PCG in the Indian population [8]. *CYP1B1* is located on chromosome 2 at position p21. The gene contains three exons and two introns. The three exons of *CYP1B1* are 371, 1,044, and 3,707 base pairs in length, and the two introns are 390 and 3032 base pairs in length. Both introns begin with the sequence GT and end with the sequence AG. The regions upstream of the 3′ end of the introns are pyrimidine rich. The coding region of *CYP1B1* starts at the 5′ end of the second exon and ends within the third exon. The putative open reading frame is 1,629 base pairs in length and codes for a 543 amino acid protein.

Although genetic heterogeneity has been reported in PCG, homogeneity in phenotype as well as genotype (p.E387K) has been reported in the Slovak Gypsy population, and common haplotypes (p.G61E, p.D374N, and p.R469W) have been associated in the Saudi Arabian population [9,10]. Confirmation of linkage between *CYP1B1* and PCG in different populations have verified the GLC3A locus (*CYP1B1*) as a major cause of PCG accounting for 85%–90% of all familial cases and 27% of sporadic cases. CYPIB1-deficient mice exhibit similar abnormalities in their trabecular meshwork similar to those in PCG patients.

**Figure 1:**
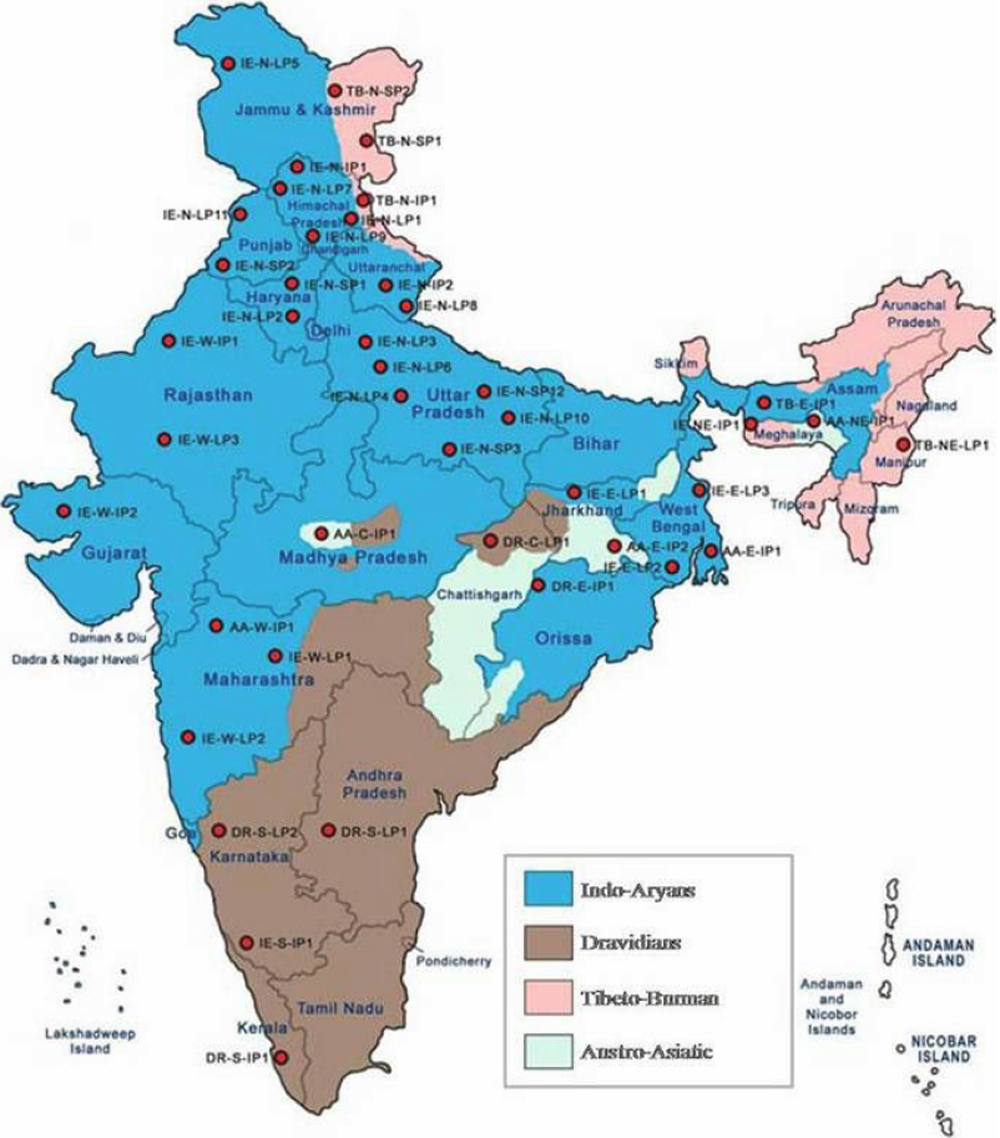
Map of India showing distribution of different ethnic populations in different parts of the country.

To date there are very few studies regarding *CYP1B1* mutations in PCG patients from northern India. India has a heterogeneous population with people from north and south India being ethnically different (Figure 1). The ethno-linguistic composition of the population of India, Pakistan, Bangladesh, Nepal, Bhutan, Maldives, and Sri Lanka mostly falls within two large groups, Dravidian and Indo-Aryan. These groups are further subdivided into numerous sub-groups, castes, and tribes. Indo-Aryans form the predominant ethno-linguistic group in Pakistan, India (the central, eastern, western, and northern regions), Nepal, Sri Lanka, and the Maldives. Dravidians form the predominant ethno-linguistic group in southern India and the northern and eastern regions of Sri Lanka [11]. To study the mutation profile of *CYP1B1* causing PCG in the north Indian population, we screened *CYP1B1* in 50 new and unrelated cases of north Indian ethnic background. The data from this preliminary study helped us to understand the common mutations prevalent in North Indian PCG patients, however larger studies are required to establish a genotype/phenotype correlation.

## Methods

### Clinical examination and selection of cases

After approval from the institutional review board (IRB # IRB00006862; All India Institute of Medical Sciences, Delhi, India), 50 consecutively diagnosed congenital glaucoma cases from northern India, presenting at the Dr. R. P. Centre for Ophthalmic Sciences (AIIMS, New Delhi, India), were enrolled for this study. The diagnosis involved clinical ocular and systemic examination. All patients underwent ophthalmoscopy and tonometry during the screening. Inclusion criteria of the patients were increased corneal diameter (>12.0 mm), raised intraocular pressure (>21 mmHg) with presence or absence of Haab’s striae and  optic disc changes (where examination was possible). Symptoms of epiphora and photophobia were the additional inclusion factors. The age of onset ranged from birth to one year. All cases were sporadic without any history of glaucoma. Fifty ethnically matched normal individuals without any history of ocular disorders were enrolled as controls. Peripheral blood samples were collected from the probands, their relatives, and controls by venipuncture after informed consent.

### Screening of *CYP1B1*

Genomic DNA was isolated from the blood and trabecular tissue by the phenol chloroform method. The DNA isolation kit (Genomic DNA Mini Kit, #GB100; Geneaid Biotech Ltd., Sijhih City, Taiwan) was used in cases with less sample (<1 ml). Mutations in *CYP1B1* are the predominant cause of PCG. The entire coding region (1.6 kb organized in exons II and III) was screened for mutations. Using three sets of overlapping primers [8], *CYP1B1* was amplified from patients and control subjects. Mutation screening was done using polymerase chain reaction-restriction fragment length polymorphism (PCR-RFLP) followed by PCR-DNA sequencing. PCR-RFLP was done for rapid detection of the six most prevalent mutations: p.G61E, p.P193L, p.Ter@223, p E229K, p.R368H, and p.R390C according to the protocol described previously [8]. DNA sequencing was done in all cases to identify all nucleotide changes like single nucleotide polymorphisms (SNPs) and mutations.

### PCR-RFLP analyses and cosegregation of mutant alleles with disease phenotype

In all RFLP-positive cases, mutations resulted in either loss or gain of restriction site. For determining the cosegregation of mutant alleles with disease phenotype in the families, the respective fragments harboring the mutation were amplified from all available family members and RFLP was done as previously described [8]. The fragments were separated on 2% agarose gel, stained with ethidium-bromide, and visualized in ultraviolet light to distinguish the wild type and mutant alleles. DNA sequencing was done to confirm the mutation.

### PCR-DNA sequencing

The whole coding region of *CYPB1* was amplified in all cases using already published primers [12,13], and amplicons were sequenced bidirectionally. The patient and control sequences were then compared to identify all mutations. The primers used were set I (1F-1R, 786 bp) [12], set II (2F-2R, 787 bp) [13], and set III (3F-3R, 885 bp) [12]. All PCRs were performed for only 30 cycles, and conditions for sets I and II were as reported earlier [12] while conditions for set III were initial denaturation at 94 °C for 3 min followed by 30 cycles each at 94 °C for 30 s, 60 °C for 30 s, and 72 °C for 1 min. PCR was done using previously described conditions [8].

## Results

### PCR-RFLP analysis of six most prevalent pathogenic mutations

PCR-RFLP analyses were performed for all six mutations (376insA, 528G→A, 923C→T, 959G→A, 1449G→A, and 1514C→T). Out of 50 patients, 21 were found positive (homozygous/heterozygous) for one or more of the above mentioned mutations. All PCR-RFLP-positive samples were subsequently sequenced to confirm the mutation. Both p.R390C and p.R390H removed the restriction site for Hin6**restriction enzyme, and DNA sequencing confirmed p.R390H in our patients, which has also been reported from Pakistan and Iran [13]. Among the six mutations screened, p.Ter@223 was the predominant disease allele in our patients and 18% of the patients were found to be heterozygous or homozygous for this mutation. The R390H mutation (16%) was the next dominant disease allele. Compound heterozygosity was observed in 10% of the cases (5/50).

### Identification of three novel missense mutations and one neutral mutation

#### Leucine24Arginine*-Novel mutation

**Figure 2:**
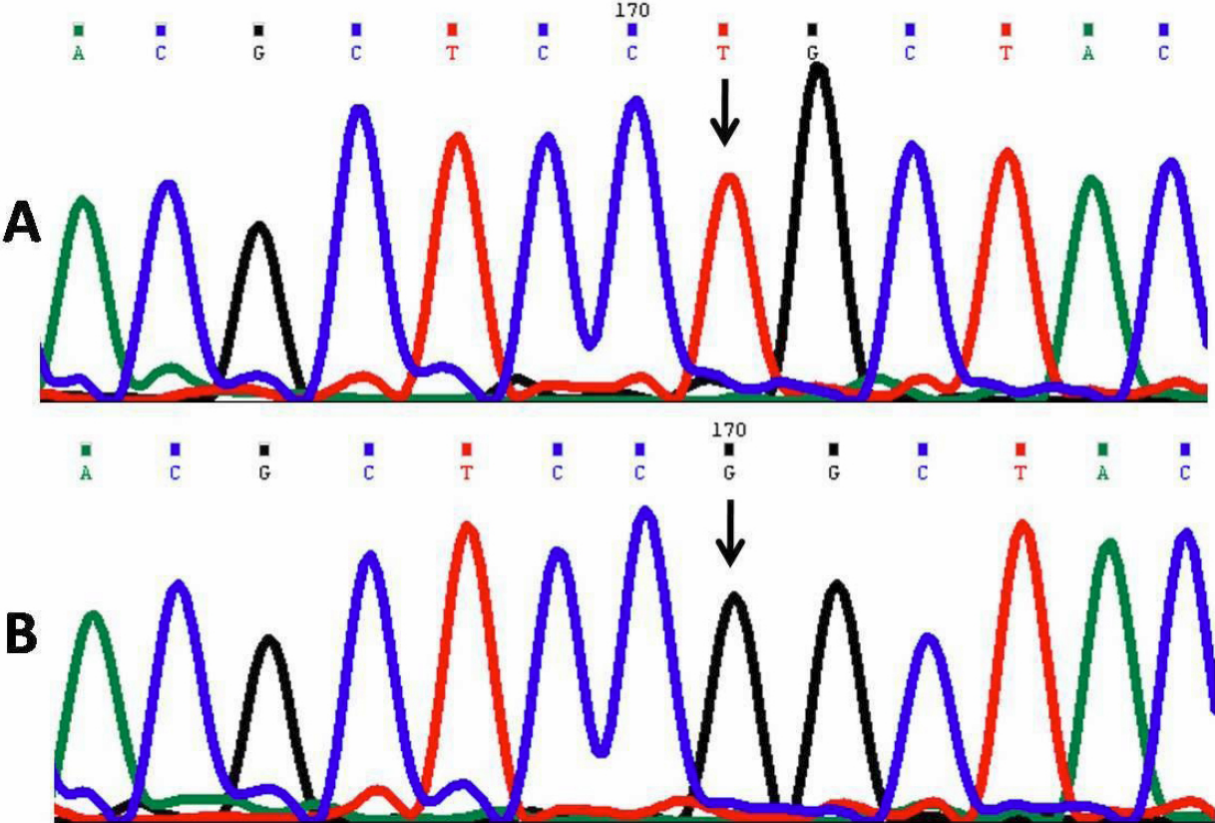
DNA sequence from exon 2 of *CYP1B1* equivalent to codon 22-26. **A**: The reference sequence derived from the control is shown. **B**: The sequence derived from PCG 022 shows the homozygous T>G mutation at genomic position 38159965, which predicts a codon change from CTG to CGG and L24R mutation.

One patient (PCG 022) harbored a novel homozygous missense mutation, p. L24R (GenBank FJ815437), in which the nucleotide thymine (T) was replaced by guanine (G) at genomic position 38159965 and nucleotide position 1432 in the gene (Figure 2). This resulted in a codon change from CTG to CGG in which a nonpolar amino acid leucine was replaced by a basic amino acid arginine at position 24 in the polypeptide.

#### Phenylalanine190Leucine*-Novel mutation

**Figure 3:**
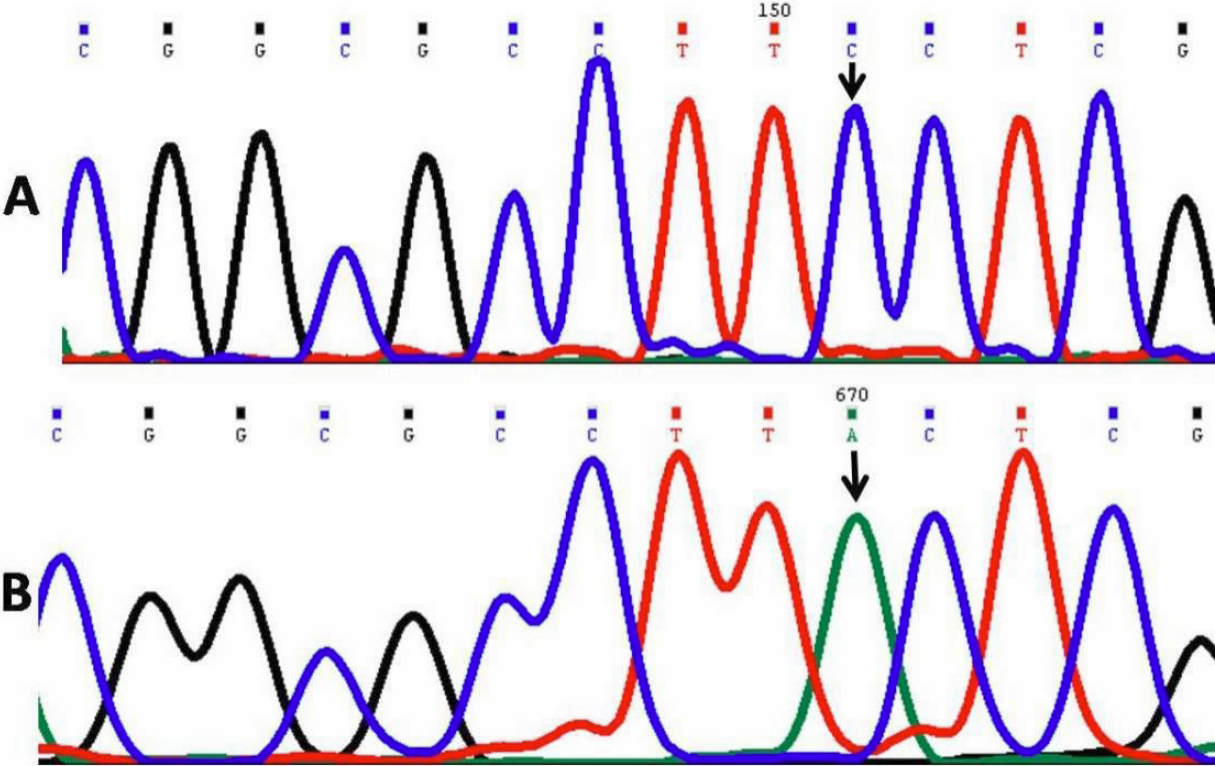
DNA sequence from exon 2 of *CYP1B1* equivalent to codon  187-191. **A**: The reference sequence derived from control is shown. **B**: The sequence derived from PCG 042 shows  the homozygous C>A mutation at genomic position 38155466, which predicts a codon change from TTC to TTA and the F190L mutation.

One patient (PCG 042) DNA sequencing revealed a change of the nucleotide cytosine (C) to adenine (A) at genomic position 38155466 and nucleotide position 1931 in the gene (Figure 3). This produced a codon change from TTC to TTA resulting in p. F190L, a novel missense mutation (GenBank FJ815438) replacing amino acid phenylalanine with leucine. This mutation was found in association with the p. R368H mutation (homozygous) in the same patient.

#### Glycine329Aspartic acid*-Novel mutation

**Figure 4:**
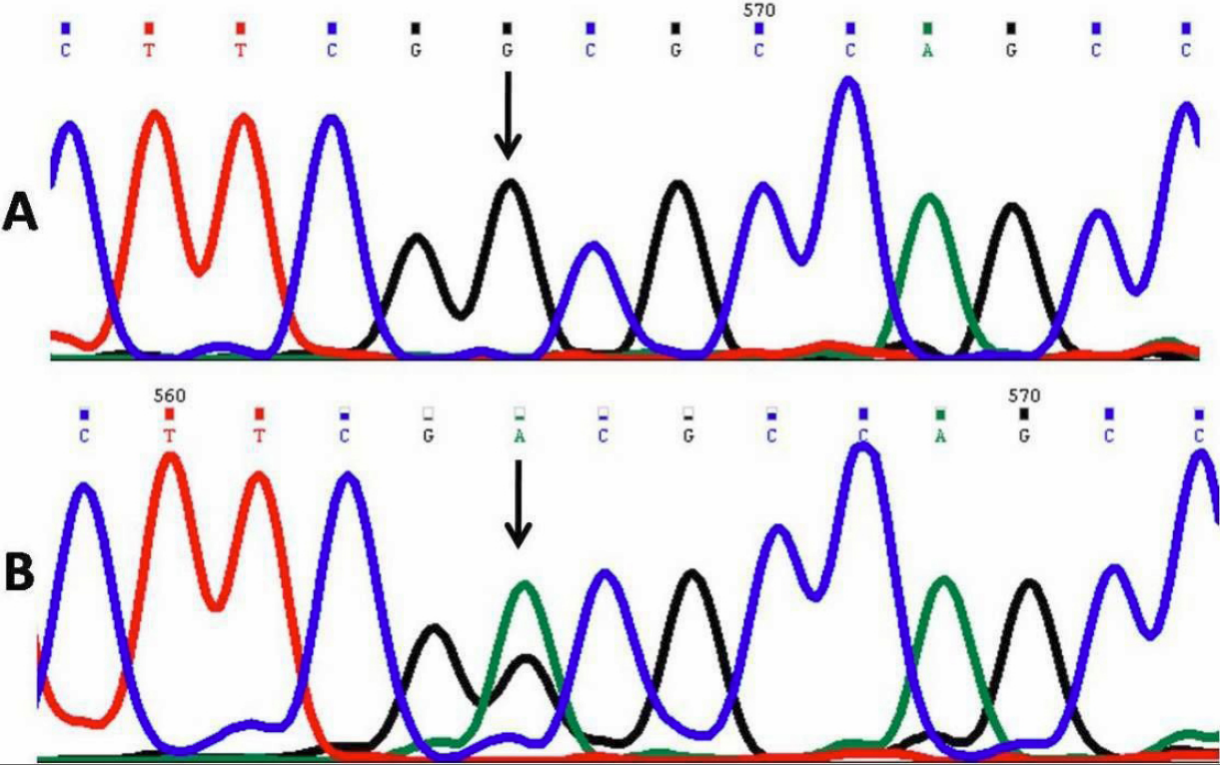
DNA sequence from exon 2nd of *CYP1B1* equivalent to codon  328-331. **A**: Reference sequence derived from control is displayed. **B**: The sequence derived from PCG 043 shows the heterozygous G>A mutation at genomic position 38155050, which predicts a codon change from GGC to GAC and the G329D mutation.

Another patient (PCG 043) DNA sequencing revealed a substitution of the nucleotide guanine (G) by adenine (A) at genomic position 38155050 and nucleotide position 2347 in the gene (Figure 4), which resulted in a codon change from GGC to GAC replacing amino acid glycine at position 329 with aspartic acid. This resulted in a novel missense mutation, p. G329D (GenBank FJ815439).

#### Leucine298Leucine

In another patient (PCG 003), cytosine (C) was substituted by thymine (T), which changed codon CTC to CTT. This resulted in a synonymous mutation with no amino acid change. Details of all mutations are given in Table 1.

**Table 1:** Details of all *CYP1B1* mutations.

**Patient ID**	**Mutation**	**Corneal Diameter (mm) OS/OD and clarity at diagnosis**	**IOP left/right eye (mmHg)**	**Buphthalmos**	**Haabs’ Striae**	**Last Cup/Disc ratio**	**Treatment**
PCG 001	R390H (H)	15x15/15x15.5; OU total corneal scarring	40/38	OU	No	Total cupping	Medical and 3XTrab/Trab OU
PCG 002	R390H (H)	13x13/13x13; OU corneal edema	36/40	OU	No	0.5:1/0.6:!	Medical and 1XTrab/Trab OU
PCG 003	R390H (H)	15x14/15x15; clear corneal	26/38	OU;R>L		Hazy cornea	Medical and 1XTrab/Trab OU; 1XTrab/Trab OS
PCG 005	R368H (H)	14.5x15/15x15; OU corneal edema	28/28	OU;L>R	No	0.7:1/0.7:1	Medical and 2XTrab/Trab OU
PCG 011	E229K (h)	14x14/14x15; clear cornea	26/22	OU	NA	NA	Medical and 1XTrab/Trab OU
PCG 012	R368H (h)	14x14.5/14x14.4	32/32	OU	OD		Medical and 1XTrab/Trab OU
PCG 013	R390H (H)	14X14/14X14	31/30	OU		Hazy media	Medical and 2XTrab/Trab OU
PCG 014	E229K (h)		25/24	OU	No	Hazy media	Medical and 2XTrab/Trab OU
PCG 015	M132R (H)	14.5X14/13.5X13	32/32	OU;L>R	No	0.6:1OU	Medical and 2XTrab/Trab OU
PCG 017	Ter@223 (H)	14X14/14X14.5	30/28	OU		NA	Medical and 2XTrab/Trab OU
PCG019	Ter@223 (h)	12X12.5/12X12; Clear cornea	22/22	OU	No	0.5:1 OU	Medical and 2XTrab/Trab OU
PCG 021	Ter@223(h)	15x16/11.5x12; OS edema	32/15	OS	OU	Hazy media	Medical and 2XTrab/Trab OS
PCG 029	Ter@223 (h)	15x15/14x14; OU edema	28/27	OU	OU	Haxy media	Medical and 2XTrab/Trab OS
PCG 022	L24R (H)*	15x15/16x16	28/28	OU;R>L	OU	0.7:1/0.5:1	Medical and 2XTrab/Trab OU
PCG 034	R390H (h) Ter@223 (h)	14x14/14x14	22/24	OD	NA	NA	Medical and 1XTrab/Trab OD
PCG 036	R390H (H) E229K (h)	15X15 OU; Clear cornea	25/26	OU	No	0.8:1 OU	Medical and 1XTrab/Trab OD
PCG 039	R368H (h) Ter@223 (h)	13x13/12x13.5; OU edema	36/34	OU	No	Near total cupping	Medical and 1XTrab/Trab OU
PCG 040	Ter@223 (h)	11.5x12.5/12x13; OU edema	23/23	OU	No	Hazy media	Medical and 1XTrab/Trab OD
PCG 041	R390H (H) Ter@223 (h)	12x10/12x12.5; OU edema	40/26	OU;R>L	No	Hazy media	Medical and 1XTrab/Trab OU
PCG 042	R368H (H) F190L (H)*	13x13/13x13; OU edema	22/24	OU	No	Hazy media	Medical and 1XTrab/Trab OU
PCG 043	R390H (h) G329D (h)*	12x12/12x12	22/26	OU	OU	0.8:1 OU	Medical and 1XTrab/Trab OU
PCG 044	Ter@223(h)	14x14/14x14	26/24	OU	No	0.8:1/0.9:1	Medical and 1XTrab/Trab OU
PCG 045	E229K (h), R390H (h)	12x12/12x12.5	20/22	OU	No	0.5:1/0.5:1	Medical and 1XTrab/Trab OU

The age of onset for all individuals was by birth. Abbreviations; H: Homozygous mutation; h: heterozygous mutation; OD-right eye; OS-left eye; OU-both eyes; X-times; Trab/Trab, combined trabeculotomy and trabeculectomy; PK-penetrating keratoplasty; NPL-no perception of light; NA-not available.

### Other previously reported mutations

#### Missense mutations

Eight patients were identified with p.R390H (five homozygous and three heterozygous), four with p.R368H (two homozygous and two heterozygous), nine with Ter@223 (one homozygous and eight heterozygous), five with p.E229K (all heterozygous) mutations, and one with a p.M132R homozygous mutation. Out of these, four patients had compound mutations (Table 1). The heterozygous p.E229K mutation was detected in five patients in our study, and Panicker et al. [8] also reported a similar mutation in heterozygous state in PCG patients.

#### Compound heterozygous mutation

Two patients (PCG 034 and PCG 041) were compound heterozygotes for R390H and Ter@223 mutations, one (PCG 039) was heterozygous for p.R368H and p.Ter@223 mutations, and two (PCG 036, 045) had p.E229K and p.R390H mutations.

### Nonpathogenic *CYP1B1* single nucleotide polymorphisms

**Figure 5:**
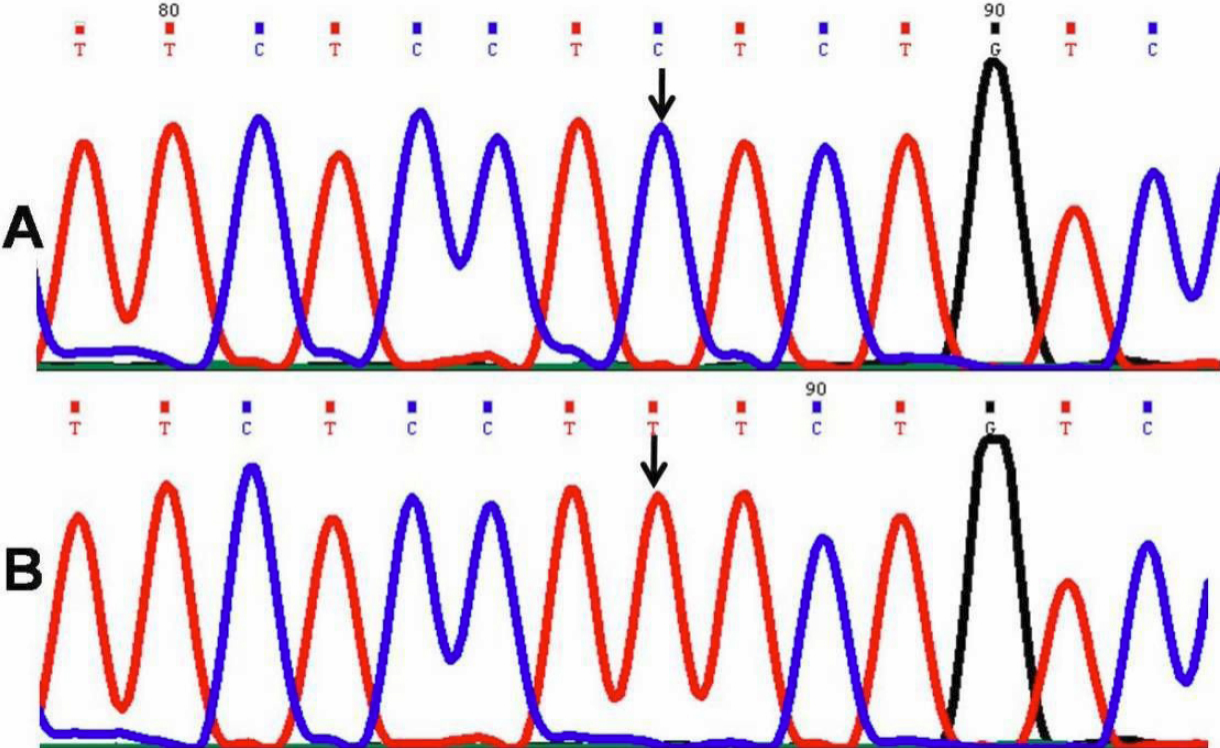
DNA sequence from intron 1 and exon 2 of *CYP1B1*. **A**: Reference sequence derived from a control is shown. **B**: DNA sequence derived from PCG 040 shows the homozygous C>T (38156048C→T) nucleotide change, an already reported polymorphism (rs2617266).

In addition to pathogenic mutations, six already reported single nucleotide polymorphisms [8] were identified in the less conserved region of *CYP1B1* both in the controls and patients. These polymorphisms were p.R48G, p.A119S, p.L432V, p.D449D, p.N453S, and rs2617266. They were revealed by DNA sequencing. rs2617266 (38156104C→T; Figure 5) was identified in two patients (PCG 039 and PCG 040). All patients with a *CYP1B1* mutation also had L432V polymorphism. These polymorphisms were present in controls.

On sequencing, pathogenic *CYP1B1* mutations were identified in 23 patients (46%) but not in controls.

### Structural and functional implications of mutant proteins

#### Leucine24Arginine (P. L24R)

**Figure 6:**
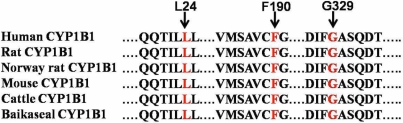
Multiple sequence alignment of various members of the cytochrome P450 super-family. **Red** letters indicate the conserved residues, which cause the congenital glaucoma phenotype when mutated.

The first 49 residues from the NH_2_-terminal end and the last 16 residues at the COOH-terminal (residues 528–543) in CYP1B1 did not have corresponding equivalent regions in CYP2c9 from where the structure of the human CYP1B1 protein was determined [14]. Leucine is conserved in the CYP1B1 protein among different species (Figure 6). Thus, we can hypothesize that leucine plays an important role in determining the structure and thus function of this protein.

#### Methionine132Arginine (M132R)

The mutation site lies in the heme-binding region (HBR), which bridges helices B’ and C. In the wild type protein, methionine extends in the interior and packs between the I-helix (between N-319 and Y-349) and one of the heme propionate groups. The amino group (NH_2_) of M-132 donates a hydrogen bond (hb) to D-326 of the I-helix and accepts a hb from G-135, W-141, and R-468, thus interconnecting the I-helix, C-helix, and heme binding loop (HBL) [15]. Replacement of methionine by arginine leads to congestion of this packing, thereby potentially harming the H-bond interaction.

#### Phenylalanine190Leucine (F190L)

The amino acid at position 190 lies between the alpha helix D and E. Phenylalanine is conserved in the CYP1B1 protein among different species (Figure 6). Phenylalanine contains an aromatic ring while leucine contains an aliphatic group. So this amino acid substitution may lead to structural alterations in the CYP1B1 protein.

#### Termination@223

In this mutation, nucleotide A is inserted in the cDNA at position 376, resulting in a frame-shift that truncates the open reading frame (ORF) by creating a premature stop codon (TGA) 636 bp downstream from the insertion site. Consequently, a 222 amino acid protein is produced, which lacks 321 amino acids from the COOH-terminal. Both the wild type and the mutant proteins contain just 10 amino acids at the NH_2_-terminus, which are similar in both, and the frame-shift eliminated all CYP1B1 domains, resulting in a functionally null allele [8].

#### Glutamic acid 229Lysine (E229K)

Position 229 lies in a key region, contributing to the three-dimensional structure of the protein [15]. This mutation occurs in the COOH-terminal of the F-helix in the vicinity of the substrate binding region (SBR). Substitution of E to K leads to a change from a negatively charged residue to a positively charged side chain and this in turn affects the local charge distribution. This mutation disturbs an important cluster of salt bridges. In wild type (WT), R-194::E-229, R-194::D-333, and D-333::K-512 form a triangle of ionic bond interactions, holding I-helix with F-helix and β-strand S3.2. Due to this mutation, the R-194::E-229 interaction is lost and has the potential to destabilize the other ionic interactions in the protein [15].

#### Glycine329Aspartic acid (G329D)

Gly329 is located in the conserved I-helix of the CYP1B1 protein in different species (Figure 6). Glycine is the smallest and most simple amino acid whereas aspartic acid contains a CH_2_COO^-^ acidic group. Replacement of glycine by aspartic acid may cause conformational changes in the protein. Ala330Phe was the first missense mutation reported in this domain [16]. This mutation represents the second missense mutation detected in this domain.

#### Arginine368Histidine (R368H)

The residue lies in between the helices J and K in an exposed loop [1,8,17]. Consequences of this change are not immediately apparent except that the positively charged amino acid arginine is replaced by histidine whose charge state depends upon its protonation state. In the WT, arginine at 368 interacts with G-365, D-367, V-363, and D-374. Because of the R368H mutation, interactions between D-367 and D-374 are weakened. How this affects the conformation and functionality of the protein is still not clear [15].

#### Arginine390Histidine (R390H)

The residue is located in the conserved alpha helix K [8]. It forms the consensus sequence, GluXXArg, which is absolutely conserved among all members of the cytochrome P450 super family [12]. Arg390 and Glu387 are one helical turn apart and are predicted to form a salt bridge. The parallel orientation of their side chains is more transparent in the three-dimensional model. Conservation of this motif indicates that it is essential for the normal function of the P450 molecule. However, the exact function is still unclear [12].

## Discussion

PCG is a clinically and genetically heterogeneous disorder. In familial cases, PCG is usually autosomal recessive. Recent genetic studies from India, Saudi Arabia, and Brazil reported several mutations in the coding region of *CYP1B1*. More than 40 different mutations have been reported in the entire coding region of *CYP1B1* [18-20], and genetic heterogeneity varies with the population. The Slovak Gypsy population showed allelic homogeneity and phenotypic uniformity [21] while other population studies reported high clinical and allelic heterogeneity. Among these groups, higher homogeneity was present in the Saudi Arabian population (with 72% having the p.G61E allele and 12% the p.R469W allele) [19] whereas other populations demonstrated increased genetic heterogeneity. High homogeneity reflects the higher rate of inbreeding in the population.

*CYP1B1* codes for a 543 amino acid protein and is expressed in the ocular tissues, in the anterior chamber of eye, and in non-ocular tissues like the kidney and liver [22]. CYP1B1 is a member of the cytochrome P450 super family of drug metabolizing enzymes. It catalyzes several oxidative reactions, some of which are biosynthetic, producing necessary hormones or compounds of intermediary metabolism in most living organisms [23]. It acts on a wide range of substrates including many xenobiotics, vitamins, and steroids. Reactions catalyzed by the enzyme include hydroxylation, sulfoxidation, N-, S-, and O-dealkylations, desulfation, deamination, and reduction of azo, nitro, and N-oxide groups [24]. It also metabolizes vitamin A in two steps to all-trans-retinal and all-trans-retinoic acid. The latter is a potent morphogen and regulates in utero development of tissue growth and differentiation [25-27]. It is involved in the metabolism of the endogenous and exogenous substrates that take part in early ocular differentiation. Any mutation in this gene can thus cause ocular development defects and result in trabecular dysgenesis as was seen in our patients on an ultrastructural study (unpublished).The same morphological alterations have been reported in CYPIB1-deficient mice [28]. Membrane-bound cytochromes such as CYP1B1 have molecular structures containing a transmembrane domain located at the NH_2_-terminal end of the molecule. This is followed by a proline-rich “hinge” region, which permits flexibility between the membrane-spanning domain and the cytoplasmic portion of the protein molecule [29]. The COOH-terminal ends are highly conserved among different members of the cytochrome P450 super family. They contain a set of conserved core structures (CCS) responsible for the heme-binding region of these molecules. The heme-binding region is essential for the normal function of every P450 molecule. Between the hinge region and the CCS lies a less conserved substrate-binding region. The cytochrome P450 protein functions like any classical enzyme molecule.

Mutations affecting such enzymes generally produce recessive phenotypes because in heterozygous subjects, the normal allele is capable of compensating for the mutant allele. Mutations in CYP1B1 interfere with the integrity of the CYP1B1 molecule as well as its ability to adopt normal conformation and to bind heme, for example, induced mutations in the hinge region have previously been reported to interfere with the heme-binding properties of the cytochrome P450 molecules [25,26]. CYP1B1 participates in the normal development and functioning of the eye by metabolizing essential molecules that are probably used in a signaling pathway [26]. Thus, normal development and differentiation of the anterior segment is important for normal ocular function, and mutations in CYPIB1 result in the malformation of anterior chamber structures, which dramatically affect visual function by blocking aqueous outflow.

*CYPIB1*  was originally identified as a dioxin-responsive cDNA clone, and its activity against various xenobiotics and pro-carcinogens has been intensively investigated [30]. A novel TCDD (2,3,7,8-tetrachlorodibenzo-p-dioxin) responsive cDNA isolated from a human keratinocyte line has been identified as a new cytochrome P450 super family member [31]. This human protein was designated as cytochrome P450 (CYP1B1). It has also been identified and cloned from mouse [32] and rat [33]. The estimated size of rodent *CYP1B1* mRNA (5.2 kb) is nearly identical to the human *CYP1B1* mRNA (5.1 kb), and each predicts a protein of 543 amino acids. CYP1B1 is constitutively expressed in the adrenals, ovary, and testis and is inducible by planar aromatic hydrocarbons, adrenocorticotropin, and peptide hormones [31,34]. It is involved in the metabolism of xenobiotics and possibly steroid hormones as suggested by its tissue distribution pattern [34,35].

In our study, 18% cases harbored the Ter@223 mutation. This mutation has been reported previously from southern India [8]. Earlier studies showed a very severe phenotype and poor prognosis with the Ter@223 mutation. In this study, one patient (PCG 017) was homozygous for the mutation, and two (PCG 034 and PCG 041) were compound heterozygotes for the Ter@223 and R390H mutations, and one patient (PCG 039) was a compound heterozygote for the Ter@223 and R368H mutations. Five patients (PCG 019, PCG 021, PCG 029, PCG 040, and PCG 044) were heterozygous for the Ter@223 mutation mutation. Congenital glaucoma is known to be an autosomal recessive disorder, but it is difficult to explain the disease pathogenesis in these five patients. Variants in both *CYPIB1* and myocilin (*MYOC*) have been detected in PCG, indicating higher complexity in the pathogenesis of this disease [36]. We have already screened these cases for mutations in *MYOC* (unpublished). These cases did not harbor any mutations in the myocilin gene. It is possible that these cases harbor some mutations at some other loci that have yet to be identified.

All patients with a *CYP1B1* mutation had the p.L432V polymorphism, and it has been reported that the CYP1B1 protein with valine at position 432 generates more free radicals and causes oxidative damage to retinal pigment epithelial cells [37]. In five patients (PCG 019, PCG 021, PCG 029, PCG 040, and PCG 044) who had the Ter@223 heterozygous mutation, we can hypothesize that in these cases, one truncated CYP1B1 protein is produced, which is a functionally null protein, and that the second allele produces a protein, which generates high reactive oxygen species (ROS) levels and thus causes oxidative stress-induced neural degeneration. Neural crest cells appear to be a particularly vulnerable cell population and are easily killed by compounds such as retinoic acid [38]. It has also been reported that developing neural crest cells are deficient in glutathione and catalase, two important antioxidant enzymes [39] that are responsible for scavenging free radicals that damage cells. Therefore, it is possible that the developing trabecular meshwork suffers oxidative damage, which may be the underlying mechanism for trabecular dysgenesis. Three patients (PCG 034, PCG 041, and PCG 039) had Ter@223 (heterozygous) with R390H and R368H mutations, and this combination may produce one functionally null protein and one defective CYP1B1 protein. All patients with Ter@223 (homozygote) and Ter@223 with p. R390H/R368H (compound heterozygote) mutations showed no perception of light even after glaucoma surgery. Little improvement in vision (light perception only) following glaucoma surgery was observed in all five cases with heterozygous Ter@223 mutation whereas other patients with homozygous/compound heterozygote *CYP1B1* mutations showed no improvement at all.

In our study, p.R390H is the second most common mutation found in 16% of patients. This mutation had been reported in other populations [13]. But p.R390H is not reported from the southern Indian population. Mutations at position 390 emerged as a hot spot for mutations in *CYP1B1*. Mutations p.R390H, p.R390C, and p.R390S have been reported from different populations [13]. Earlier studies showed that prognosis of patients with a p.R390H mutation is poorer than with a p.R390C mutation. Patients with the p. R390H mutation showed little or no improvement in vision compared to patients with the p.R390C mutation. Patients with the p.R368H homozygous mutation had no light perception while one patient with the R368H heterozygous mutation showed little improvement in vision following surgery.

One patient (PCG 022) was a 10-month-old girl from a non-consanguineous Hindu family who harbored a p.L24R mutation. She had bilateral buphthalmos with raised intraocular pressure (IOP) in both eyes (left; 24 mmHg and right; 30 mmHg). The corneal diameter was 15 mm x 15 mm in the left eye and 16 mm x 16 mm in the right eye with a cup:disc ratio of 0.5:1 and 0.6:1 in the left and right eye, respectively. She underwent bilateral trabeculotomy and trabeculectomy twice within a period of four months and is able to fix onto and follow light.

Three-dimensional models from *Bacillus magaterium* (CYP108 and CYP102 protein) were used in earlier studies to determine the structure of the CYP1B1 protein, and these studies showed that the first 49 amino acids were not conserved in the protein from the cytochrome 450 families [14]. Multi-alignment of different CYP1B1 proteins showed that leucine is conserved at position 24 and may play a significant role in protein functioning [Figure 1]. Another patient (PCG 022) with the *CYP1B1* p.L24R mutation only had bilateral buphthalmos with raised intraocular pressure. It is possible that p.L24R is a pathogenic mutation that shows improvement post-surgery and thus has a good prognosis, though further functional studies are required to ascertain the pathogenic role of the p. L24R mutation.

Patient (PCG042) harbored p.F190L mutation, was a five-day-old child from a non-consanguineous Muslim family with bilateral buphthalmos at birth with raised IOP (22 mmHg in both eyes) and corneal diameter of 13 mm x 13 mm with severe bilateral corneal edema. Fundus examination was not possible because of the corneal edema. This patient was heterozygous for the  p.R368H (homozygous) and p.F190L mutations. Due to compound heterozygous status, a defective CYP1B1 protein is produced, and the patient showed no improvement in vision following surgery.

Another patient (PCG 043) had congenital glaucoma at birth with IOPs of 22 and 26 mmHg in the right and left eye, respectively, with corneal diameter of 12 mm x 12.5 mm with presence of Haab’s striae in both eyes. She was a compound heterozygote for the p.G329D (heterozygous) and p.R390H (heterozygous) mutations. It has been previously reported [13] that the p.R390H mutation produces a defective CYP1B1 protein as the glycine residue is conserved at this position in the alpha I helix, thus a nucleotide change at this locus can create a defective protein. Since congenital glaucoma is an autosomal recessive disorder and both mutations are heterozygous in this case, we can say that the combination of both mutations produces a defective protein, which is unable to perform its function. Hence, we can say p.G329D is a pathogenic mutation.

In our study, 46% of patients harbored *CYP1B1* mutations. This is slightly higher than the southern Indian population (43.5%) but lower than the Slovak gypsy population (100%) [7,21]. Both p.G61E and p.P193L mutations were not present in our population while these were present in congenital glaucoma patients from southern India [8]. We found no significant differences between patients with homozygous *CYP1B1* mutations and patients with compound heterozygote *CYP1B1* mutations. However, patients with heterozygous *CYP1B1* mutations had a better prognosis after surgery than patients with homozygous/compound heterozygous *CYP1B1* mutations.

The mutation spectrum of northern Indian congenital glaucoma patients is totally different from the mutation spectrum of southern Indian patients. The most prevalent mutation, p. R368H, in the southern population is present in only 8% (4/50) of our patients while other mutations like p.Ter@223 and p.R390H emerged as the most prevalent *CYP1B1* mutation in northern Indian patients. This difference in the mutation spectrum of both the populations may be explained on the basis of different ethnicity of both populations (Figure 1). The northern Indian population is predominantly Aryan population while the southern Indian population is Dravidian with totally different morphological phenotype and genetic background.

Three novel mutations were identified in this study. Studies of pathogenic sequence variants of *CYP1B1* in different populations may contribute to a better insight into the molecular pathogenesis of congenital glaucoma. The new mutations found in our study as well as those already reported could also add to the existing repertoire of the SNPs, which can be used in the future as diagnostic or prognostic markers for detection and to chart the path of disease progression. This will further help in the elucidation of the structure-function relationship of *CYP1B1* and hence may lead to the development of novel therapeutics in management of congenital glaucoma in familial cases.
